# Thiosulfate sulfurtransferase prevents hyperglycemic damage to the zebrafish pronephros in an experimental model for diabetes

**DOI:** 10.1038/s41598-022-16320-1

**Published:** 2022-07-15

**Authors:** Zayana M. Al-Dahmani, Xiaogang Li, Lucas M. Wiggenhauser, Hannes Ott, Paul D. Kruithof, Sergey Lunev, Fernando A. Batista, Yang Luo, Amalia M. Dolga, Nicholas M. Morton, Matthew R. Groves, Jens Kroll, Harry van Goor

**Affiliations:** 1 Department of Pharmacy and Drug Design, University of Groningen, Groningen, The Netherlands; 2 Department of Vascular Biology and Tumor Angiogenesis, European Center for Angioscience (ECAS), Medical Faculty Mannheim, Heidelberg University, 68167 Mannheim, Germany; 3 Department of Pathology and Medical Biology, University Medical Center Groningen, Groningen, The Netherlands; 4 Department of Pharmacy, Molecular Pharmacology, University of Groningen, Groningen, The Netherlands; 5 Centre for Cardiovascular Science, The Queen’s Medical Research Institute, University of Edinburgh, Edinburgh, UK; 6 Department of Medical Microbiology and Infection Prevention, University of Groningen, University Medical Center Groningen, 9700 RB Groningen, The Netherlands; 7 XB20 Drug Design, Groningen Research Institute of Pharmacy, University of Groningen, 9700 AD Groningen, The Netherlands

**Keywords:** Proteins, Mitochondrial proteins, Biochemistry, Chemical biology

## Abstract

Thiosulfate sulfurtransferase (TST, EC 2.8.1.1), also known as Rhodanese, was initially discovered as a cyanide detoxification enzyme. However, it was recently also found to be a genetic predictor of resistance to obesity-related type 2 diabetes. Diabetes type 2 is characterized by progressive loss of adequate β-cell insulin secretion and onset of insulin resistance with increased insulin demand, which contributes to the development of hyperglycemia. Diabetic complications have been replicated in adult hyperglycemic zebrafish, including retinopathy, nephropathy, impaired wound healing, metabolic memory, and sensory axonal degeneration. Pancreatic and duodenal homeobox 1 (Pdx1) is a key component in pancreas development and mature beta cell function and survival. *Pdx1* knockdown or knockout in zebrafish induces hyperglycemia and is accompanied by organ alterations similar to clinical diabetic retinopathy and diabetic nephropathy. Here we show that *pdx1*-knockdown zebrafish embryos and larvae survived after incubation with thiosulfate and no obvious morphological alterations were observed. Importantly, incubation with hTST and thiosulfate rescued the hyperglycemic phenotype in *pdx1*-knockdown zebrafish pronephros. Activation of the mitochondrial TST pathway might be a promising option for therapeutic intervention in diabetes and its organ complications.

## Introduction

Type 1 and type 2 diabetes are pathological disorders defined by improper glucose, lipid, and protein metabolism as a result of defective insulin secretion or action^1^. Glucose serves as precursor for various metabolic pathways. GLUT4, the glucose transporter protein, is responsible for glucose uptake in insulin-dependent tissues such as adipose tissues and muscles. GLUT1, the other insulin-independent transporter, is widely expressed in most tissues^2^. Insulin, which is released by the pancreas, is essential for glucose homeostasis. Hyperglycemia, inflammation, obesity, and other conditions cause insulin resistance or decreased insulin effects in target tissues^3^. When peripheral tissues develop insulin resistance, the pancreas generates more insulin to counteract the condition. Apoptotic cell death occurs when the β -cells fail to adapt for the stress, resulting in a deficiency in insulin synthesis and secretion. Hyperglycemia arises as a result of this effect, which steadily raises blood glucose levels^2^.

The zebrafish can be used to study metabolic diseases because of its high conservation of lipid metabolism, pancreas structure, adipose biology and glucose homeostasis^4–6^. The zebrafish’s pronephron is comprised of a duct, a tubule and a glomerulus that develop in a stepwise fashion and begin to function as a blood filter in zebrafish at 48 h post fertilization (hpf)^7^. Blood glucose levels in adult zebrafish and zebrafish embryos can be altered using a variety of techniques. Exposing zebrafish to high concentration of glucose is the simplest technique to raise blood and tissue glucose. In young and adult zebrafish, incubation of the animals in a high glucose medium for two months simulates immediate and chronic hyperglycemia^8,9^. However, more advanced methods of hyperglycemia production are required to obtain the multifactorial and complex metabolic characteristics encountered in T1DM and T2DM patients, such as; diet-based methods, chemical methods, genetic methods, and hybrid methods^9,10^. The efficiency in which zebrafish can be genetically modified is the main advantage for their application to diabetes research. The ability to introduce targeted mutations using sequence specific transcription activators like effector nucleases (TALENs) or the clustered regularly interspaced short palindromic repeats (CRISPR) system has made the zebrafish a highly attractive model for studying the consequences of loss of function alleles^11–13^. The implications of the null mutation of pdx1 (pancreatic and duodenal homeobox 1) in zebrafish were investigated after the successful formation of the Zebrafish Mutation Project^14–17^. Homozygous *pdx1* mutations lead to disruption of pancreatic islet development and glucose homeostasis^15^. Adult zebrafish develop organ complications following prolonged diabetic conditions, with the pdx1 zebrafish mutant being a novel model organism showing hyperglycemia-induced activation of retinal angiogenesis^14,16,18^.

Thiosulfate Sulfurtransferase (TST, Rhodenase), is a mitochondrial enzyme originally discovered as a cyanide detoxifying enzyme in 1933. It can convert cyanide to the less toxic thiocyanate^19–21^. TST also maintains the native architecture of reconstituted iron-sulfur proteins by mobilizing sulfur for iron-sulfur cluster formation or repair^22^. TST is critical in the metabolism of sulfide (H_2_S) by sulfur dioxygenase and the degradation of reactive oxygen species (ROS)^23^. The latter role places TST in the mitochondrial sulfide oxidation pathway, where it catalyzes the transfer of sulfane sulfur from glutathione persulfide (GSSH) to sulfite, producing thiosulfate^24,25^. TST is among few peripherally expressed genes that is established as a positive genetic factor in metabolic health, showing a distinct negative association with the development of obesity related insulin resistant type 2 diabetes^26,27^.

Thiosulfate, the substrate for mitochondrial thiosulfate sulfurtransferase (TST), is used clinically as remedy for cyanide poisoning and as an off-label medication in the treatment of calciphylaxis^28^. Oral administration of STS in N-u-nitro-L-arginine (L-NNA) induced hypertensive rats improved renal function and hemodynamics. Treatment with thiosulfate in addition to lisinopril improved renal vascular resistance (RVR) and glomerular damage, implying that additional mechanisms could be involved^29^. Moreover, thiosulfate is suggested to restore endothelium homeostasis by enhancing nitric oxide synthase activity and restoring GSH through thiosulfate sulfurtransferase (TST) activity. High concentration of dissolved thiosulfate in tissue causes inhibition of the mitochondrial cytochrome complex IV and generates sulfide-induced oxidative stress^30^. This contrasts with its capacity to alleviate oxidative stress at lower concentrations, as TST may feed sulfur and reducing equivalents to antioxidant systems^27^.

Mechanistically, thiosulfate reacts with reactive thiol (R-SH) in the body nonenzymatically to generate H_2_S and oxidized thiol (R-S-S-R). The reduction in the end-diastolic and end-systolic diameters of the left ventricle in experimental heart disease were accomplished by scavenging oxidant radicals and producing H_2_S which may have worked as an antioxidant by boosting glutathione levels. Thiosulfate serves as an antioxidant in the cardiac tissue, scavenging super oxide, thereby aiding in the rescue of the failing heart^31^. Thiosulfate enhances systolic function and reduces hypertension, left ventricular hypertrophy, fibrosis, and systemic oxidative stress in the same way that angiotensin converting enzyme (ACE) inhibition does^32^. These findings suggest that taking thiosulfate orally has therapeutic promise in the treatment of hypertensive heart disease.

Administration of thiosulfate, a substrate for TST, ameliorates glucose intolerance and insulin resistance in diabetic mice, in part by increasing secretion of the insulin-sensitizing hormone adiponectin from adipocytes^26^3T3-L1^27,33^. Morton et al.^26^ found similar evidence for beneficial metabolic effects of TST stimulation in human adipose tissue. This followed the discovery that variance at the murine *Tst* gene underpinned a major quantitative trait locus for healthy leanness in mice that was driven by an adipose-selective increase in *Tst* expression^26^. Thus, TST had a strong negative correlation with fat mass and plasma glucose levels, while having a positive correlation with adiponectin expression^26,27,34^. In the present paper we study whether TST and TST activation through its main substrate thiosulfate, can rescue hyperglycemia induced kidney damage the renal system in zebrafish embryos.

## Materials and methods

### Expression and purification of human thiosulfate sulfurtransferase (hTST)

A synthetic gene for human TST codon-optimized for E. coli (Eurofins) was cloned into a pETM11 vector (EMBL) and confirmed by Sanger sequencing (pETM11-hTST)^35^. BLR (DE3) competent cells (Novagen) were transformed using the pETM11-hTST expression plasmid and a glycerol stock was prepared and frozen at − 80 °C for further use. This stock was used to inoculate 20 ml LB media supplemented with 100 mg/L kanamycin and 2 mM magnesium chloride. After overnight incubation at 37 °C this culture was used to inoculate 2 L of TB media supplemented with 100 mg/L kanamycin and 2 mM magnesium chloride, which was induced with 0.4 mM IPTG at OD600 ≈ 0.8. The cultures were grown overnight at 18 °C. The cultures were harvested at 4556 g for 15 min. Pellets were resuspended in Lysis buffer (50 mM TRIS pH 8.0, 25 mM NaCl, 10% v/v glycerol, 5 mM dithiothreitol), which were supplemented with DNAse1 and Lysozyme for 30 min. Subsequently, the resuspended pellets were sonicated on ice and the lysate clarified by centrifugation at 41,000 g for 45 min. hTST protein was initially purified by 5 ml His-trap column (GE) using medium pressure liquid chromatography system (NGC, BioRad). The column was washed with lysis buffer supplemented with 10–30 mM Imidazole followed by elution using the Lysis buffer supplemented with 150–250 mM Imidazole and the eluted fractions were assessed by SDS-gel electrophoresis. Elution fractions containing approximately 70–80% pure hTST were pooled and the sample buffer was rapidly exchanged against freshly prepared Cation exchange buffer A (20 mM Na-citrate pH 5.0, 10 mM NaCl, 10 mM DTT) using buffer exchange/desalting column (G25 resin, GE). The hTST sample was purified using cation-exchange chromatography using a MonoS (GE Healthcare) column equilibrated in Cation exchange buffer A and eluted on a linear gradient with buffer A supplemented with 1 M NaCl. Fractions containing hTST were again identified using SDS-PAGE prior to concentration and final polishing using a size-exclusion chromatography (SEC) using HiLoad 16/60 Superdex 75 column (GE Healthcare) equilibrated with SEC buffer (100 mM Hepes, 150 mM NaCl, 10% v/v Glycerol at pH 7.3) on the automated NGC chromatography system (BioRad). The hTST was eluted as a single peak at an elution volume of approximately 70 ml. This peak was pooled and concentrated to 18 mg ml^-1^ by using a centrifuge concentration unit (Sartorius) and stored in 50% (v/v) glycerol a − 80 °C for further use.

### Zebrafish lines and husbandry

All experimental procedures on animals were approved by Medical Faculty Mannheim (license no.: I-19/01) and carried out in accordance with the approved guidelines. Embryos of the *Tg(wt1b:EGFP)*^17^ line were raised and staged as described according to hours post fertilization (hpf)^36^. Embryos were kept in egg water at 28.5 °C with 0.003% 1-phenyl-2-thiourea (Sigma) to suppress pigmentation. Adult zebrafish were kept under a 13 h light − 11 h dark cycle and fed with living shrimps and fish flake food.

### Incubation of zebrafish eggs with thiosulfate

Approximately 20–23 fertilized eggs were incubated in a 6-well plate with 4 to 5 mL solutions that were changed daily. Solutions contained egg water, thiosulfate (10 mM, 1 mM, 0.1 mM, 0.01 mM and 0) and 0.003% 1-phenyl-2-thiourea.

### Injection of TST and morpholinos into zebrafish embryos

The sequence of morpholino oligonucleotides were: SB-Pdx1-Mo: 5’-GAT AGT AAT GCT CTT CCC GAT TCA T-3’ (targets the zebrafish Pdx1 translation start site); Control-MO: 5’-CCT CTT ACC TCA GTT ACA ATT TAT A-3′^36^.

Both Pdx1 and control morpholinos were diluted to 6 µg/µL in 0.1 M KCl. hTST was diluted to 13.5 µg/µL and 1 µg/µL in 0.1% BSA/PBS. One nanoliter of morpholino or hTST [1 µg/µl] was injected into the yolk sac of one cell or two cell stage embryos^16,17,37^.

### Microscopy and analysis of pronephric alterations

To analyse pronephric structures of embryos, 48 hpf old *Tg (wt1b: EGFP)* embryos were anesthetized with 0.003% tricaine and mounted in 1% low melting point agarose (Promega), dissolved in egg water, in a dorsal manner. Images were taken using a Leica DFC420 C camera, attached to a Leica MZ10 F modular stereo microscope. Alterations of pronephros were quantified by measuring the glomerular length, width and neck size using the Leica LAS V4.8 software^17^.

### Statistics

Statistical significance between different groups was analyzed using 2-sided unpaired Student’s t-test. Results are expressed as mean ± SD. *P* values < 0.05 were considered as significant: * < 0.05, ** < 0.01, and *** < 0.001.

## Results

### Thiosulfate does not alter pronephros development in zebrafish embryos

**Figure 1:**
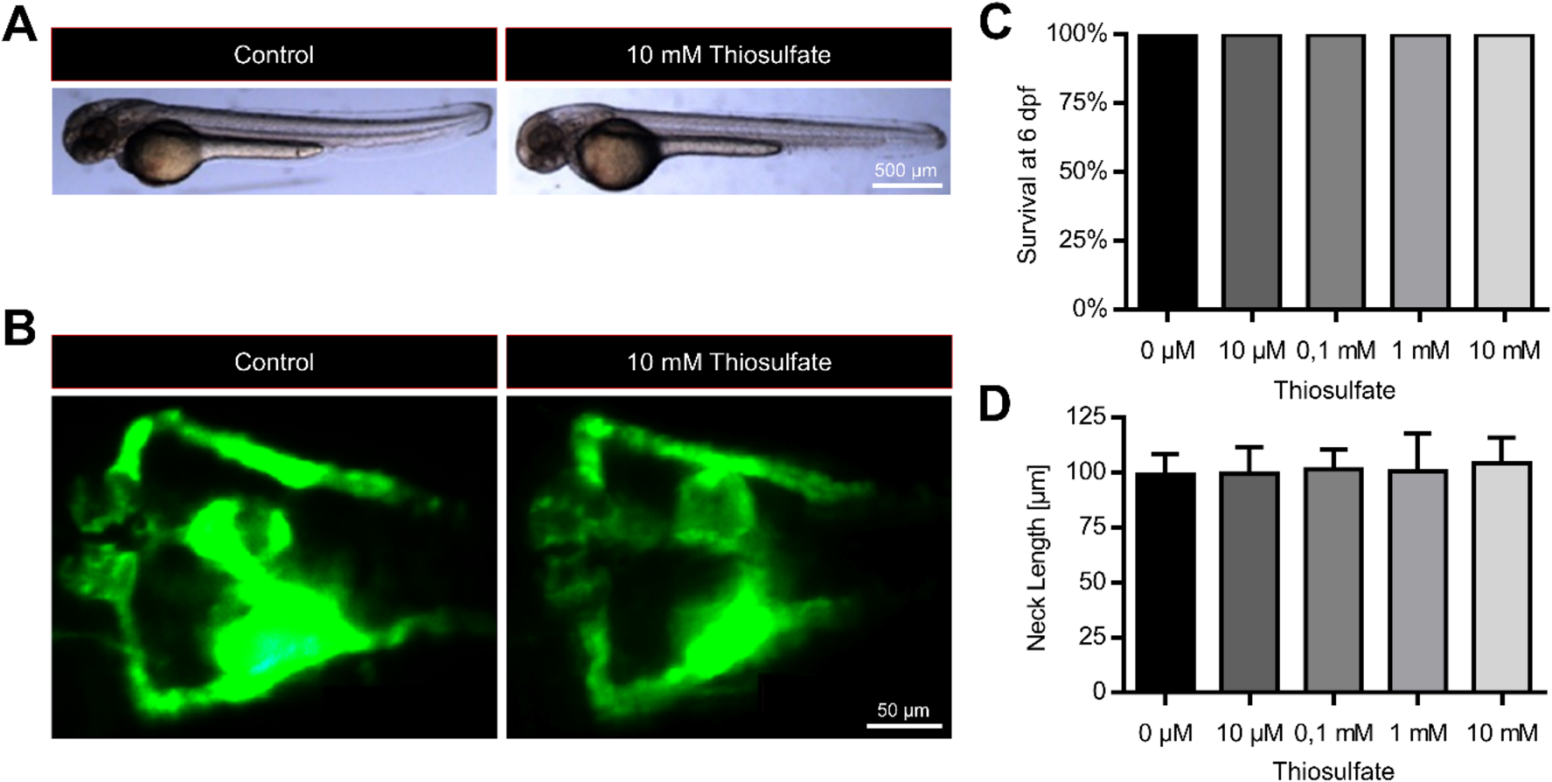
Thiosulfate exposure does not affect viability or pronephros morphology in healthy zebrafish larvae. (**A**) Representative overview of the zebrafish appearance at 48 hpf with and without 10 mM thiosulfate. Zebrafish embryos exposed to 10 mM thiosulfate did not show any gross morphological changes compared to controls. (**B**) Representative visualization of the pronephros phenotype in *Tg(wt1b:EGFP)* embryos at 48 hpf with and without 10 mM thiosulfate. Thiosulfate did not have any visible effect on the pronephros morphology compared to controls. (**C**) Evaluation of the survival in zebrafish larvae exposed to thiosulfate for 6 days. Thiosulfate did not lead to any detrimental effects regarding survival in any of the studied concentrations (n = 15 for each condition). (**D**) Evaluation of the pronephric neck length in *Tg(wt1b:EGFP)* embryos at 48 hpf exposed to different thiosulfate concentrations. Thiosulfate did not significantly alter the pronephric neck length in healthy zebrafish larvae (at least 10 larvae per group were analyzed). Scale bars are 500 μm in (**A**) and 50 μm in (**B**). Statistics utilized one-way ANOVA + Sidak’s post-hoc (**D**); mean + SD reported; *p < 0.05.

To test a potential therapeutic benefit of thiosulfate on hyperglycemia-induced kidney alterations in zebrafish embryos, we initially tested increasing concentrations of thiosulfate on viability and gross morphology of zebrafish embryos and larvae over the range of 0.01 to 10 mM for 6 days. As shown in Fig. 1a, all embryos and larvae survived after thiosulfate incubation and we could not observe obvious morphological alterations (Fig. 1c and d). For morphological analysis of the embryonic kidney (pronephros), we took advantage of the transgenic zebrafish line *Tg(wt1b: EGFP)* which shows a pronephros-specific EGFP expression, labelling the glomerulus and the tubular structures that together form the pronephros. Subsequently, we incubated the zebrafish embryos in different concentrations of thiosulfate, and at 48 hpf the glomerular length, width and the tubular structures showed no alterations (Fig. 1b,E and f) indicating no effect of thiosulfate on the zebrafish pronephros.

### Thiosulfate and hTST have beneficial effects on hyperglycemia induced kidney damage in zebrafish embryos

**Figure 2:**
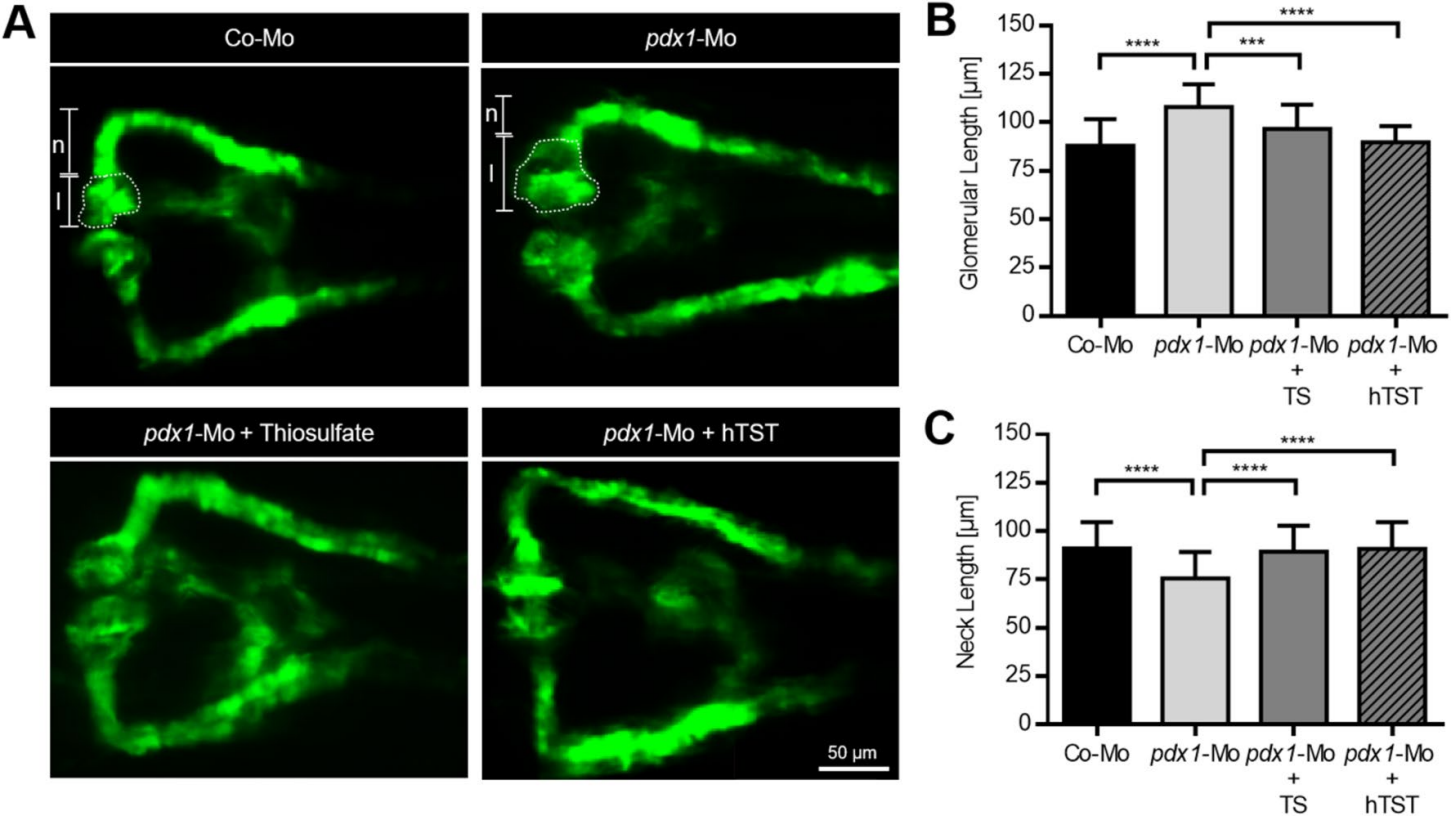
Thiosulfate and human thiosulfate sulfurtransferase treatment rescues pdx1 morpholino-induced hyperglycemic pronephros damage. (**A**) Representative visualization of the zebrafish pronephros in Tg(wt1b:EGFP) embryos at 48 hpf with different experimental conditions. Embryos injected with only a control morpholino (Co-Mo) show the typical pronephros morphology at 48 hpf, while injection with the pdx1 morpholino (pdx1-Mo) leads to a hyperglycemia-mediated pronephros phenotype with an increased glomerular length and a shortened pronephric neck length. Treatment via either exposure to 10 mM thiosulfate (TS) or injection of 1 nl human thiosulfate sulfurtransferase (hTST) at a concentration of 1 µg/µL rescued the pronephros phenotype in the pdx1 morphants. (**B**–**C**) Evaluation of the pronephros phenotype in Tg(wt1b:EGFP) embryos at 48 hpf shows significant rescue of both (**B**) glomerular length and (**C**) pronephric neck length by TS and hTST treatment in pdx1 morphants (n = 45 for Co-Mo and pdx1-Mo, n = 30 for the TS and hTST groups). Measurements for glomerular length (l) and pronephric neck length (n) have been marked and the glomerulus was encircled for one pronephric side in two images in (**A**) as example. Lengths given in (**B**–**C**) represent the total sum of both the left and right side measurements added up. The scale bar is 50 μm in (A). Statistics utilized one-way ANOVA + Sidak’s post-hoc (**B**–**C**); mean + SD reported; ***p < 0.001, ****p < 0.0001.

To test whether thiosulfate and hTST could rescue alterations of the pronephros caused by hyperglycemia, we induced hyperglycemia in zebrafish embryos utilizing a knockdown strategy targeting the transcription factor Pancreatic and duodenal homeobox 1 (Pdx1), which is essential for pancreatic cell development, using the morpholino technology^31^. Compared with the control group (Fig. 2a and b), the *pdx1* morphants displayed an enlarged glomerulus at 48 hpf, where the length was significantly increased from 88 µm in the controls to 108 µm in *pdx1* morphants. The pronephric neck was significantly shortened to 76 µm in *pdx1* morphants compared with 91 µm in the control group (Fig. 2a and c). The structural alterations represented by glomerular enlargement and reduced pronephric neck length in *pdx1* morphants were almost entirely normalized upon hTST injection (Fig. 2a and e), and beneficially restored by thiosulfate incubation (Fig. 2a and d).

## Discussion

Zebrafish models of human disease are key screening tools in drug discovery research as they allow identification of novel pharmacological targets by linking gene function with pathogenesis. The development of novel genome editing techniques and renal reporter lines make zebrafish an attractive model in biomedical research, including renal and diabetes studies^38^. The pronephros of zebrafish consists of two glomeruli, which fuse at the embryonic midline and are connected by pronephric tubules to the bilateral pronephric ducts^39^. This simple structure of pronephros in zebrafish and its rapid development make it a useful model for investigating the renal morphology and function in the context of a developing organism^39^. In this study, hyperglycemic kidney damage in zebrafish embryos induced by *pdx1* knockdown caused alterations of the pronephros. Incubation with thiosulfate and the injection of hTST recovered these structural alterations. This report supports TST and thiosulfate capacity to rescue hyperglycemia induced kidney damage in zebrafish, without being direct regulators of glucose homeostasis—as has been previously reported in rats^26,33^.

Administration of hTST as well as administration of the substrate of TST (thiosulfate) in zebrafish phenotypically rescued damage caused by hyperglycemia. This reduction of diabetic damage in the zebrafish could arise from multiple mechanisms. For example, hyperglycemia results in kidney damage partly through an increase in reactive oxygen species (ROS) which are generated endogenously in the process of mitochondrial oxidative phosphorylation^23,40^. Carcinogenesis, neurodegeneration, atherosclerosis, diabetes, and aging have all been linked to oxidative stress, which causes direct or indirect ROS mediated damage to nucleic acids, proteins, and lipids^41^. Since TST can provide reducing equivalents to glutathione and thioredoxin^42^, and may aid in the constitution of selenocysteine proteins^27,43^, TST activity may also reduce oxidative stress as a result of hyperglycemia, although this hypothesis remains unproven. Moreover, TST could also be a link between two major mitochondrial systems that would have an impact on diabetic symptoms. TST may influence respiratory capacity by interacting with iron-sulfur centres in the electron transport chain^27^. Improved mitochondrial function has been linked to improved cellular health, including maintained glucose uptake in adipocytes and enhanced secretion of insulin sensitizing factors such as adiponectin^26^, that subsequently mediate improved glucose tolerance, indicating a strong link between TST and glucose intolerance^25,27^. TST can supply electrons into antioxidant systems as detailed above^27^. Since inflammation appears to play a major role in diabetes, enhanced antioxidant activity would be crucial for counteracting the metabolic abnormalities associated with obesity. Finally, increased mitochondrial respiration suggests an increase in metabolite consumption, which directly reduced cellular hyperglycemia. Taken together, our results also support the use of zebrafish as a screening model for drugs targeting diabetic nephropathy.

## Conclusions

Our results show that thiosulfate and hTST have beneficial effects on hyperglycemia induced kidney damage in zebrafish embryos. TST is involved in several mechanisms that may significantly reduce oxidative stress as a result of hyperglycemia. TST can interact with iron-sulfur centres in the electron transport chain which could improve the respiratory capacity and mitochondrial function. Increased mitochondrial respiration implies increased metabolite consumption, which directly reduces intracellular glucose levels. In conclusion, this data provides a strong indication that TST could reduce diabetic damage in zebrafish and other diabetic models.

## Data Availability

**T**he data presented in this study are available on request from the corresponding author. The data are not publicly available for privacy reasons.

## References

[1] Gioacchini FM (2018). Hyperglycemia and diabetes mellitus are related to vestibular organs dysfunction: Truth or suggestion? A literature review. Acta Diabetol..

[2] Giri B (2018). Chronic hyperglycemia mediated physiological alteration and metabolic distortion leads to organ dysfunction, infection, cancer progression and other pathophysiological consequences: An update on glucose toxicity. Biomed. Pharmacother..

[3] Ebeling P, Koistinen HA, Koivisto VA (1998). Insulin-independent glucose transport regulates insulin sensitivity. FEBS Lett..

[4] Zang L, Maddison LA, Chen W (2018). Zebrafish as a model for obesity and diabetes. Front. Cell Dev. Biol..

[5] Elo B, Villano CM, Govorko D, White LA (2007). Larval zebrafish as a model for glucose metabolism: Expression of phosphoenolpyruvate carboxykinase as a marker for exposure to anti-diabetic compounds. J. Mol. Endocrinol..

[6] Wiggenhauser LM, Kroll J (2019). Vascular damage in obesity and diabetes: Highlighting links between endothelial dysfunction and metabolic disease in Zebrafish and Man. Curr. Vasc. Pharmacol..

[7] Drummond IA (1998). Early development of the zebrafish pronephros and analysis of mutations affecting pronephric function. Development.

[8] Connaughton VP, Baker C, Fonde L, Gerardi E, Slack C (2016). Alternate immersion in an external glucose solution differentially affects blood sugar values in older versus younger zebrafish adults. Zebrafish.

[9] Heckler K, Kroll J (2017). Zebrafish as a model for the study of microvascular complications of diabetes and their mechanisms. Int. J. Mol. Sci..

[10] Salehpour A, Rezaei M, Khoradmehr A, Tahamtani Y, Tamadon A (2021). Which hyperglycemic model of zebrafish (Danio rerio) Suites My Type 2 diabetes mellitus research? A scoring system for available methods. Front. Cell Dev. Biol..

[11] Hwang WY, Peterson RT, Yeh JRJ (2014). Methods for targeted mutagenesis in zebrafish using TALENs. Methods.

[12] Shah AN, Davey CF, Whitebirch AC, Miller AC, Moens CB (2015). Rapid reverse genetic screening using CRISPR in zebrafish. Nat. Methods.

[13] Lodd, E. *et al.* The combination of loss of glyoxalase1 and obesity results in hyperglycemia. *JCI Insight* **4**, (2019). 10.1172/jci.insight.126154 PMC6629122 31217350

[14] Middel CS, Hammes HP, Kroll J (2021). Advancing diabetic retinopathy research: Analysis of the neurovascular unit in zebrafish. Cells.

[15] Kimmel RA (2015). Diabetic pdx1-mutant zebrafish show conserved responses to nutrient overload and anti-glycemic treatment. Sci. Rep..

[16] Wiggenhauser LM (2020). Activation of retinal angiogenesis in hyperglycemic pdx1-/- zebrafish mutants. Diabetes.

[17] Wiggenhauser LM (2022). pdx1 knockout leads to a diabetic nephropathy– like phenotype in zebrafish and identifies phosphatidylethanolamine as metabolite promoting early diabetic kidney damage. Diabetes.

[18] Ali Z (2020). Photoreceptor degeneration accompanies vascular changes in a zebrafish model of diabetic retinopathy. Investig. Ophthalmol. Vis. Sci..

[19] Aminlari M, Malekhusseini A, Akrami F, Ebrahimnejad H (2007). Cyanide-metabolizing enzyme rhodanese in human tissues: Comparison with domestic animals. Comp. Clin. Path..

[20] Chaudhary M, Gupta R (2012). Cyanide detoxifying enzyme: Rhodanese. Curr. Biotechnol. e.

[21] Chow SF, Horowitz PM (1985). Spectral differences between rhodanese catalytic intermediates unrelated to enzyme conformation. J. Biol. Chem..

[22] Cipollone R, Ascenzi P, Visca P (2007). Common themes and variations in the rhodanese superfamily. IUBMB Life.

[23] Nandi DL, Horowitz PM, Westley J (2000). Rhodanese as a thioredoxin oxidase. Int. J. Biochem. Cell Biol..

[24] Westley J (2006). Rhodanese. Adv. Enzymol. Relat. Areas Mol. Biol..

[25] Hildebrandt TM, Grieshaber MK (2008). Three enzymatic activities catalyze the oxidation of sulfide to thiosulfate in mammalian and invertebrate mitochondria. FEBS J..

[26] Morton NM (2016). Genetic identification of thiosulfate sulfurtransferase as an adipocyte-expressed antidiabetic target in mice selected for leanness. Nat. Med..

[27] Kruithof PD (2020). Unraveling the role of thiosulfate sulfurtransferase in metabolic diseases. Biochim. Biophys. Acta Mol. Basis Dis..

[28] Peng T (2018). Systematic review of sodium thiosulfate in treating calciphylaxis in chronic kidney disease patients. Nephrology.

[29] Nguyen ITN (2020). Sodium thiosulfate improves renal function and oxygenation in L-NNA–induced hypertension in rats. Kidney Int..

[30] Jiang J (2016). Hydrogen sulfide-mechanisms of toxicity and development of an antidote. Sci. Rep..

[31] Sen U (2008). Cardioprotective role of sodium thiosulfate on chronic heart failure by modulating endogenous H2S generation. Pharmacology.

[32] Nguyen ITN (2021). Cardiac protection by oral sodium thiosulfate in a rat model of L-NNA-induced heart disease. Front. Pharmacol..

[33] Lainšček D, Šuštar U, Carter RN, Morton NM, Horvat S (2020). Tst gene mediates protection against palmitate-induced inflammation in 3T3-L1 adipocytes. Biochem. Biophys. Res. Commun..

[34] Yanai H, Yoshida H (2019). Beneficial effects of adiponectin on glucose and lipid metabolism and atherosclerotic progression: Mechanisms and perspectives. Int. J. Mol. Sci..

[35] Dümmler A, Lawrence AM, de Marco A (2005). Simplified screening for the detection of soluble fusion constructs expressed in E. coli using a modular set of vectors. Microb. Cell Fact..

[36] She J, Yuan Z, Wu Y, Chen J, Kroll J (2018). Targeting erythropoietin protects against proteinuria in type 2 diabetic patients and in zebrafish. Mol. Metab..

[37] Jörgens K (2015). High tissue glucose alters intersomitic blood vessels in zebrafish via methylglyoxal targeting the VEGF receptor signaling cascade. Diabetes.

[38] Drummond I (2003). Making a zebrafish kidney: A tale of two tubes. Trends Cell Biol..

[39] Wingert RA, Davidson AJ (2008). The zebrafish pronephros: A model to study nephron segmentation. Kidney Int..

[40] Dan Dunn J, Alvarez LAJ, Zhang X, Soldati T (2015). Reactive oxygen species and mitochondria: A nexus of cellular homeostasis. Redox Biol..

[41] Ray PD, Huang BW, Tsuji Y (2012). Reactive oxygen species (ROS) homeostasis and redox regulation in cellular signaling. Cell. Signal..

[42] Isom GE, Way JL (1984). Effects of oxygen on the antagonism of cyanide intoxication: Cytochrome oxidase, in vitro. Toxicol. Appl. Pharmacol..

[43] Ogasawara Y, Lacourciere G, Stadtman TC (2001). Formation of a selenium-substituted rhodanese by reaction with selenite and glutathione: Possible role of a protein perselenide in a selenium delivery system. Proc. Natl. Acad. Sci. U. S. A..

